# P01-006 – MEFV mutation detection in Arabic patients

**DOI:** 10.1186/1546-0096-11-S1-A10

**Published:** 2013-11-08

**Authors:** R Taha, S Ayesh, M Kambouris, H El-Shanti

**Affiliations:** 1 Shafallah Medical Genetics Center, Doha, Qatar; 2 Gene Medical Labs, Gaza, Palestinian Territory, Occupied

## Introduction

Autoinflammatory diseases are a group of disorders characterized by seemingly unprovoked inflammation in the absence of high-titer autoantibodies or antigen-specific T-cells. Familial Mediterranean fever (FMF) is the archetypal hereditary periodic fever syndrome and autoinflammatory disorder. It is characterized by recurrent self-limiting episodes of fever and painful polyserositis. FMF is an autosomal recessive disorder, with considerable prevalence in specific ethnic groups, namely, non-Ashkenazi Jews, Armenians, Turks and Arabs and the FMF carrier rate can be as high as one in four. The gene responsible for FMF, *MEFV*, is located on the short arm of human chromosome 16, and was independently identified by two positional cloning consortia. Mutations, as well as, polymorphisms in *MEFV* are continuously identified. In Arabic FMF patients the spectrum and distribution of *MEFV* mutations are distinctive and the portion of unidentified mutations is undoubtedly the highest amongst the groups commonly affected by FMF. The comprehensive identification of *MEFV* mutant alleles among FMF patients is needed for the efficient examination of specific genotype – phenotype correlation patterns and for the development of molecular tools to support the clinical diagnosis.

## Objectives

To identify *MEFV* mutations in a cohort of Arabic patients using a comprehensive approach that could identify coding and non-coding variations, large duplications or deletions, as well as intronic variations.

## Methods

We obtained 100 patients of Palestinian origin with clear FMF symptomatology consistent with the clinical diagnostic criteria and for whom only one mutation has been identified. We applied a comprehensive mutation analysis approach that involves sequencing of exons and splice sites, sequencing putative regulatory regions, Multiplex Ligation-dependent Probe Amplification (MLPA) technique to detect large deletions or duplications, and sequencing of the entire genomic sequence of MEFV.

## Results

We did not identify any mutations by sequencing *MEFV* exons and splice sites, as well as putative regulatory regions. Similarly, MLPA did not reveal any large deletions or duplications within the genomic sequence of MEFV. The sequencing of the entire genomic sequence identified 20 different rare intronic variants that were each identified in 1-3 affected individuals only, and were not identified in about 700 ethnically matched control chromosomes. The biological significance of these variations could not be determined.

## Conclusion

It has been suggested that the reduced identification of *MEFV* mutant alleles in Arabic patients is due to the lack of application of a comprehensive mutation detection methodology. However, the current study negates that hypothesis. We hypothesize that the current sequencing technology produces a preferential amplification of one allele over the other due to extensive polymorphism within the genomic sequence. To examine this hypothesis we developed a parallel sequencing approach for the genomic *MEFV* sequence. At this stage of the study, we could not exclude the effect of modifier genes or any other loci that influence the clinical picture of FMF in Arabic populations.

## Disclosure of interest

None declared

